# Supplementary material for: Predicting visual function by interpreting a neuronal wiring diagram
Source: Nature. 2024 Oct 2;634(8032):113–23. doi: 10.1038/s41586-024-07953-5 (PMC11446822; doi:10.1038/s41586-024-07953-5)
Supplement: Supplementary file 6 — CRF and ERF predictions for individual TmY4 and TmY9 cells. Analogous to Supplementary Data 3, but for TmY target types. Shown are the top four monosynaptic pathways, the strongest pathway passing through each of the top ten intermediary types (ranking from Extended Data Fig. 7), and the trisynaptic pathway Tm1–TmY–Dm3–TmY (see the section entitled Prediction of spatial normalization). [file 41586_2024_7953_MOESM6_ESM.zip › DataS4/TmY9qΓèÑ/720575940634174233.pdf]

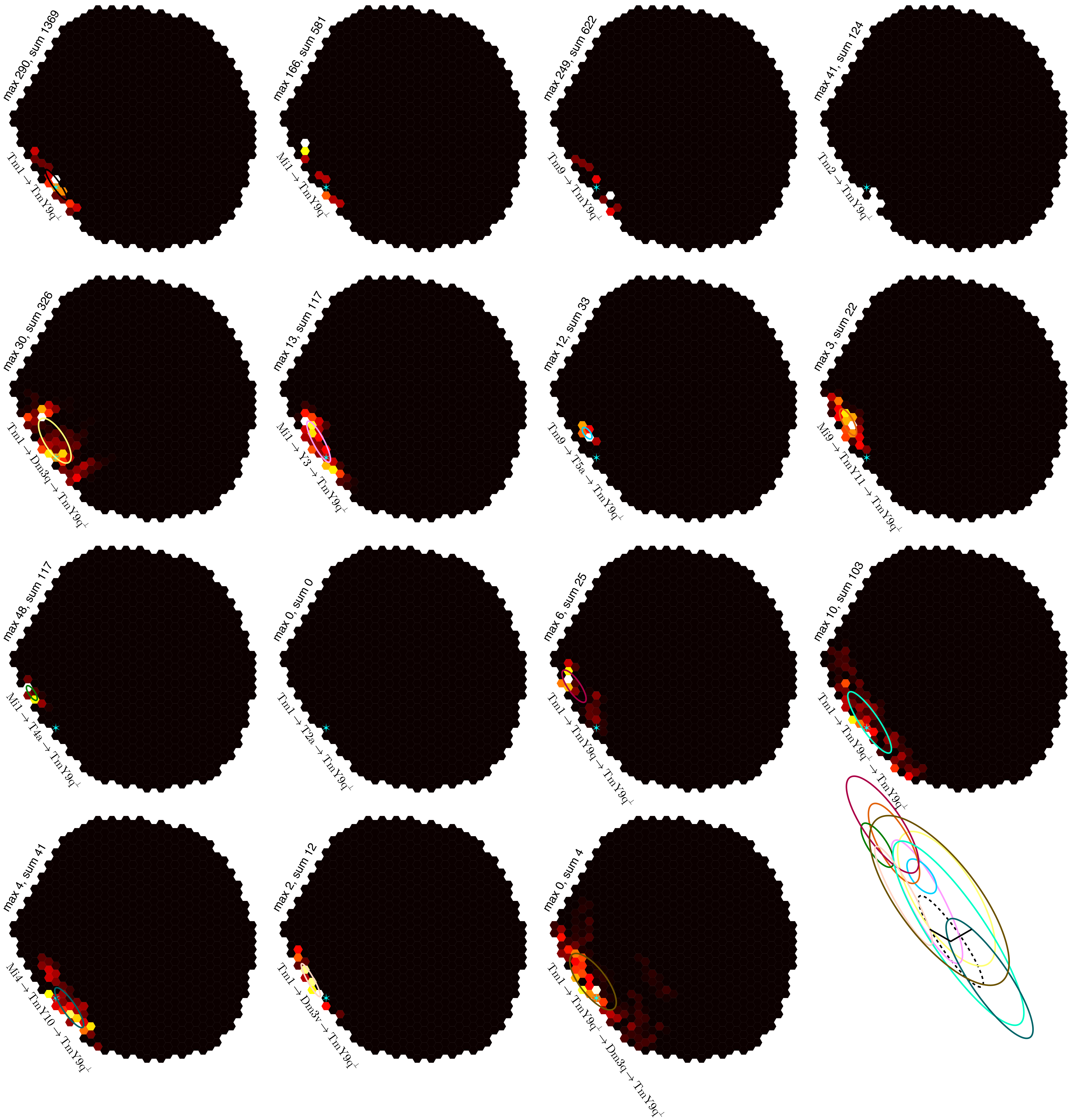

max 290, sum 1369
Tm1 → TmY9q⊥
max 166, sum 581
Mi1 → TmY9q⊥
max 249, sum 622
Tm9 → TmY9q⊥
max 41, sum 124
Tm2 → TmY9q⊥
max 30, sum 326
Tm1 → Dm3q → TmY9q⊥
max 13, sum 117
Mi1 → Y3 → TmY9q⊥
max 12, sum 33
Tm9 → T5a → TmY9q⊥
max 3, sum 22
Mi9 → TmY11 → TmY9q⊥
max 48, sum 117
Mi1 → T4a → TmY9q⊥
max 0, sum 0
Tm1 → T2a → TmY9q⊥
max 6, sum 25
Tm1 → TmY9q → TmY9q⊥
max 10, sum 103
Tm1 → TmY9q⊥ → TmY9q⊥
max 4, sum 41
Mi4 → TmY10 → TmY9q⊥
max 2, sum 12
Tm1 → Dm3v → TmY9q⊥
max 0, sum 4
Tm1 → TmY9q⊥ → Dm3q → TmY9q⊥
